# Outcome of cardiac surgery in patients with congenital heart disease in England between 1997 and 2015

**DOI:** 10.1371/journal.pone.0178963

**Published:** 2017-06-19

**Authors:** Aleksander Kempny, Konstantinos Dimopoulos, Anselm Uebing, Gerhard-Paul Diller, Ulrich Rosendahl, George Belitsis, Michael A. Gatzoulis, Stephen J. Wort

**Affiliations:** 1 Adult Congenital Heart Centre and National Centre for Pulmonary Hypertension, Royal Brompton Hospital, Imperial College London, London, United Kingdom; 2 Adult Congenital and Valvular Heart Disease Centre, Department of Cardiology and Angiology, University Hospital Muenster, Muenster, Germany; 3 Department of Cardiac Surgery, Royal Brompton Hospital, Imperial College London, London, United Kingdom; 4 Department of Cardiac Surgery, Great Ormond Street Hospital, London, United Kingdom; Bambino Gesù Children's Hospital, ITALY

## Abstract

**Background:**

The number of patients with congenital heart disease (CHD) is increasing worldwide and most of them will require cardiac surgery, once or more, during their lifetime. The total volume of cardiac surgery in CHD patients at a national level and the associated mortality and predictors of death associated with surgery are not known. We aimed to investigate the surgical volume and associated mortality in CHD patients in England.

**Methods:**

Using a national hospital episode statistics database, we identified all CHD patients undergoing cardiac surgery in England between 1997 and 2015.

**Results:**

We evaluated 57,293 patients (median age 11.9years, 46.7% being adult, 56.7% female). There was a linear increase in the number of operations performed per year from 1,717 in 1997 to 5,299 performed in 2014. The most common intervention at the last surgical event was an aortic valve procedure (9,276; 16.2%), followed by repair of atrial septal defect (9,154; 16.0%), ventricular septal defect (7,746; 13.5%), tetralogy of Fallot (3,523; 6.1%) and atrioventricular septal defect (3,330; 5.8%) repair. Associated mortality remained raised up to six months following cardiac surgery. Several parameters were predictive of post-operative mortality, including age, complexity of surgery, need for emergency surgery and socioeconomic status. The relationship of age with mortality was “U”-shaped, and mortality was highest amongst youngest children and adults above 60 years of age.

**Conclusions:**

The number of cardiac operations performed in CHD patients in England has been increasing, particularly in adults. Mortality remains raised up to 6-months after surgery and was highest amongst young children and seniors.

## Introduction

Survival prospects have considerably improved in patients with congenital heart disease (CHD) over the past few decades, mainly due to earlier diagnosis and ongoing improvement of surgical and percutaneous procedures performed in this population [1]. While surgery performed at a younger age is mostly life saving, cardiac surgery performed later on in life is often elective and performed to improve symptoms or prognosis. In these cases accurate estimation of mortality associated with cardiac surgery remains crucial for informed consent; it provides an overview on the balance between estimated risks and benefits of surgery and, thus, improves decision-making. Several tools have been developed to estimate the surgical risk. These include, depending on the age of patient or type of procedure, the Aristotle Score, Society of Thoracic Surgery–European Association for Cardio-Thoracic Surgery (STAT) Congenital Heart Surgery Mortality score, Risk Adjustment for Congenital Heart Surgery 1 score (RACHS-1), EuroSCORE or EuroSCORE II [2–7]. These scores, although based on solid data and often externally validated, account for mortality related to surgery only if patients either die within the same hospital stay or within 30 days after surgery. Patients, however, may die after discharge, in another hospital or beyond 30 days after surgery, but still due to complications associated with the procedure. Moreover, many percutaneous interventions have been successfully introduced over the past two decades and may have changed the volume and type of cardiac surgery performed and may have, therefore, changed the overall surgical mortality due to selection of patients. Therefore, we sought to (1) assess the volume of cardiac surgery in CHD patients in England, (2) to evaluate the associated mortality as well as the temporal distribution of deaths after surgery and (3) to compare the actual mortality after surgery to the one that the risk scores account for.

## Methods

We performed a retrospective analysis of the hospital episode statistics database for England from 1997 to 2015. Each patient under the National Health Service (NHS) in England is assigned a unique number, which enables tracking of patient events within the electronic system. Patients admitted to hospitals have multiple data recorded, including the first twenty most relevant diagnoses for each hospital episode, as well as data on the type and time of treatment provided. Diagnoses are coded using the 10^th^ revision of the International Classification of Diseases system (ICD-10), while procedures are reported using the ‘Office of Population Censuses and Surveys’ system (OPCS-4). Anonymized data are available for research purposes and access to the database was granted for this project by the Health and Social Care Information Centre. We identified all patients diagnosed with any CHD (ICD-10 codes ‘Q2x.x’) who underwent cardiac surgery between 1997 and 2015, with the exception of heart or heart and lung transplant. If a patient underwent more than one cardiac surgery, the last one was included in the survival analysis and analysis of post-operative hospital stay and type of procedures were performed, as presented in tables. Surgery was classified as complex if at least two procedures were at the time of surgery. Data on mortality and cause of death were retrieved for all patients from the UK Office for National Statistics.

### Statistical analysis

Statistical analyses were performed using R-package version 3.2.2 for Windows (R Foundation for Statistical Computing, Vienna, Austria) [8]. Tables were formatted using Microsoft Excel for Windows, version 2013. Continuous variables are presented as median with interquartile range (IQR) in square brackets. Categorical variables are presented as number and percentage. Comparison between groups was performed for continuous variables using Mann-Whitney test while distribution of categorical variables was assessed using chi-squared test. Kaplan–Meier method was used for assessing survival following cardiac surgery in the entire population and in subgroups. Standardized mortality ratio and expected mortality were estimated using an age and gender-matched cohort from the Interim Life Tables for England and Wales published by the Government Actuary’s Department [9]. Differences in survival were assessed using log-rank test. Centile curves for the relationship between age and the length of hospital stay after surgery were generated using a combined method based on the Rigby and Stasinopoulos algorithm using the GAMLSS package for R. [10–13] The relationship of absolute mortality risk at 6 months after surgery and age was assessed using a generalized additive model. The relationship of independent variables and 6-month mortality after surgery was assessed using logistic regression. A sensitivity analysis was performed by excluding surgery involving CABG or mitral valve procedure. A two-sided p-value of <0.05 was considered indicative of statistical significance.

## Results

**Fig 1:**
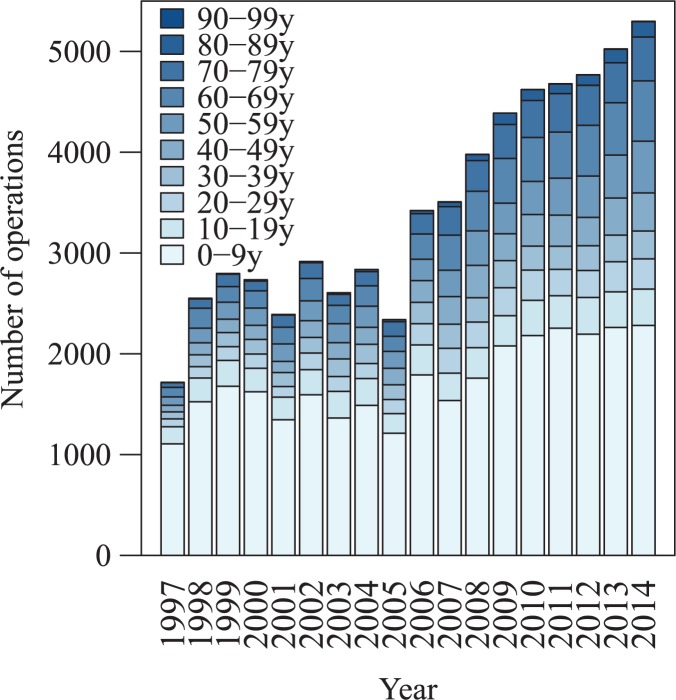
Number of cardiac operations in patients with CHD by year and age. There was an increase over time in the number of annually performed cardiac surgery in CHD patients, irrespective of age; the highest increase was observed in adults.

Overall 57,293 patients with CHD were identified, who underwent 63,811 cardiac surgical procedures between 1997 and 2015. There was a linear increase in the number of surgical procedures performed per year, with 1,717 performed in 1997 and 5,299 performed in 2014, giving an annual increment of 191.3 [95% CI of 160.4–222.1] per year (Fig 1, S1 Fig). The majority of patients (51,821; 90.4%) underwent one cardiac surgery during the study period, while 4,581 (8.0%) underwent two and 891 (1.6%) had three or more cardiac operations. Mean age at the time of last cardiac surgery was 11.9 [1.0–51.3] years and the distribution of age was bimodal, with the first peak for patients aged 0–5 years and the second one for 60–65 years olds; 46.7% were adult (>16years). The majority of patients were female (56.7%, Table 1).

**Table 1:** Baseline characteristics, surgical era and post-operative mortality.

				Mode of surgery			
		All surgery		Elective		Non elective	
Parameter	Unit	n = 57,293		n = 42,412		n = 14,881	
**Age at surgery**	Years	11.9 [1.0–51.3]		16.9 [2.2–53.0]		1.0 [0.3–43.6]	
0-4y	n / %	23,935	41.8%	15,049	35.5%	8,886	59.7%
5-9y	n / %	3,790	6.6%	3,351	7.9%	439	3.0%
10-19y	n / %	4,395	7.7%	3,800	9.0%	595	4.0%
20-39y	n / %	6,542	11.4%	5,535	13.1%	1,007	6.8%
40-59y	n / %	8,294	14.5%	6,652	15.7%	1,642	11.0%
60y≤	n / %	10,337	18.0%	8,025	18.9%	2,312	15.5%
**Female gender**	n / %	32,485	56.7%	23,399	55.2%	9,086	61.1%
**Ethnicity**							
White	n / %	40,331	70.4%	30,417	71.7%	9,914	66.6%
Asian	n / %	3,591	6.3%	2,616	6.2%	975	6.6%
Black	n / %	1,435	2.5%	1,026	2.4%	409	2.7%
Other or not stated	n / %	11,936	20.8%	8,353	19.7%	3,583	24.1%
**Surgery time period**							
1997–1999	n / %	6,183	10.8%	4,590	10.8%	1,593	10.7%
2000–2004	n / %	11,895	20.8%	9,072	21.4%	2,823	19.0%
2005–2009	n / %	15,839	27.6%	11,772	27.8%	4,067	27.3%
2010–2015	n / %	23,376	40.8%	16,978	40.0%	6,398	43.0%
**Follow-up time**	Years	6.2 [2.8–11.3]		6.6 [3.0–11.6]		5.4 [2.1–10.3]	
**Cumulative follow-up time**	Years	415,323		317,496		97,826	
**Mortality after surgery**							
30d or same hospital stay	(%)	2.93%	1680	1.65%	698	6.60%	982
6-months mortality observed	(%)	4.23% (4.06–4.23)		2.42% (2.28–2.57)		9.36% (8.89–9.83)	
6-months mortality expected*	(%)	0.29%		0.28%		0.32%	

This table presents data for the last cardiac surgery performed in each patient. (*) Expected mortality was calculated using age and gender matched UK population. Observed 6-months mortality was assessed using the Kaplan Meier method.

### Type of surgical procedures

Table 2 presents a summary of surgical procedures performed. Out of the 57,293 most recent procedures performed in each patient, 41,305 (72.1%) were single procedures, not involving any other major cardiac surgical intervention, while 15,988 (27.9%) were complex and involved at least one additional intraoperative cardiac surgical intervention (S2 Fig). The majority of surgery was elective (42,412; 74%; S3 Fig), with a median time between booking the surgery and hospital admission of 61.0 [21.0–120.0] days. The most common type of cardiac surgery was an aortic valve procedure (9,276 cases in total; 16.2% of all procedures) and included aortic valve replacement using a mechanical prosthesis (4,170; 45.0%), xenograft (3,759; 40.5%), allograft (269; 2.9%) or aortic valve repair (467; 5.0%). In 611 (6.6%) the type of prosthesis was not specified. The second most common procedure was atrial septal defect (ASD) repair (9,154 cases; 16.0%), followed by ventricular septal defect (VSD) repair (7,746; 13.5%), tetralogy of Fallot repair (3,523; 6.1%) and atrioventricular septal defect (AVSD) repair (3,330; 5.8%).

**Table 2:** Surgery classified by main procedure performed.

	All surgery		Single procedure		Combined procedure	
Type of cardiac surgery	n	%	n	%	n	%
Aortic valve repair or replacement	9,276	16.2%	7,538	81.3%	1,738	18.7%
ASD repair	9,154	16.0%	8,218	89.8%	936	10.2%
VSD repair	7,746	13.5%	5,142	66.4%	2,604	33.6%
ToF repair	3,523	6.1%	2,964	84.1%	559	15.9%
AVSD repair	3,330	5.8%	1,927	57.9%	1,403	42.1%
Mitral valve repair or replacement	3,272	5.7%	2,198	67.2%	1,074	32.8%
						
CABG using mammary arteries	2,860	5.0%	306	10.7%	2,554	89.3%
TGA repair, arterial switch	2,651	4.6%	1,170	44.1%	1,481	55.9%
Pulmonary valve repair or replacement	2,272	4.0%	1,905	83.8%	367	16.2%
CABG using veins	1,602	2.8%	356	22.2%	1,246	77.8%
Aortic root replacement	1,466	2.6%	1,101	75.1%	365	24.9%
TAPVC repair	957	1.7%	655	68.4%	302	31.6%
						
Tricuspid valve repair or replacement	820	1.4%	498	60.7%	322	39.3%
TCPC	556	1.0%	537	96.6%	19	3.4%
AP Fontan circulation	532	0.9%	494	92.9%	38	7.1%
DORV repair	214	0.4%	65	30.4%	149	69.6%
TGA repair, atrial switch	175	0.3%	88	50.3%	87	49.7%
Other	6,887	12.0%	6,143	89.2%	744	10.8%
Sum	57,293	100.0%	41,305	72.1%	15,988	27.9%

This table presents data for the last cardiac surgery performed in each patient. Single procedure—if no additional surgical cardiac procedure was performed during the surgery; Combined procedure—if any additional surgical cardiac procedure was performed during the surgery; ASD, atrial septal defect; VSD, ventricular septal defect; ToF, tetralogy of Fallot; AVSD, atrioventricular septal defect; CABG, coronary artery bypass graft; TGA, transposition of the great arteries; TAPVC, total anomalous pulmonary venous connection; TCPC, total cavopulmonary connection; AP Fontan circulation, atrio-pulmonary connection; DORV, double outlet right ventricle; TGA arterial switch—arterial switch for TGA or double switch for 'congenitally corrected' TGA.

The most common cardiac surgery performed in children (30,543 operations, 53.3% of total) was VSD closure (7,089; 23.2% of all surgery performed in children), followed by ASD closure (4,924; 16.1%), tetralogy of Fallot repair (3,351; 11.0%) and AVSD repair (2,723; 8.9%). The majority of cardiac surgery in children involved only one surgical procedure (23,012; 75.3%).

### Post-surgical hospital stay and mortality

The median post-operative stay was 7.0 [IQR 5.0–11.0] days and was significantly lower in patients undergoing elective surgery, compared to the patients undergoing non-elective surgery (7.0 [5.0–9.0] vs. 9.0 [6.0–16.0] days, respectively, P<0.001). The likelihood of undergoing non-elective surgery in adults increased with age (OR 1.11 per 10 years, 95%CI 1.10–1.13, P<0.001).

**Fig 2:**
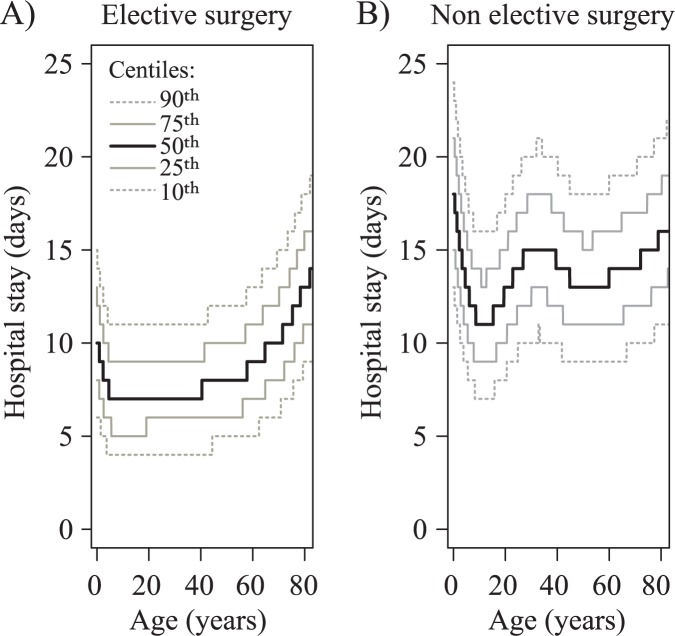
Length of hospital stay after cardiac surgery in CHD patients. Length of hospital stay after surgery in CHD patients undergoing (A) elective or (B) non-elective cardiac surgery.

The length of post-operative stay was age-dependent for elective surgery. It was shortest for patients aged 5–40 years (6.0 [5.0–8.0] days) and was longer both in children below 5 years (7.0 [5.0–12.0] days, P<0.001, with post-operative stay decreasing with age), and in patients above 40 years (7.0 [6.0–11.0], P<0.001, with hospital stay increasing further with age in this group; Fig 2).

**Fig 3:**
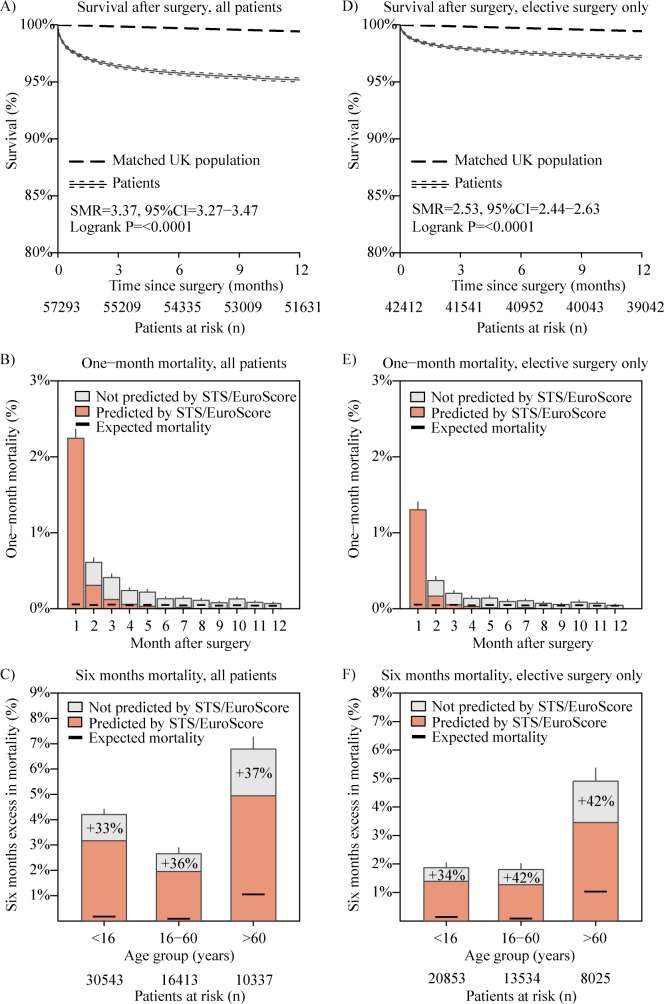
Survival after cardiac surgery in CHD patients. This panel presents figures for all surgical procedures (A, B, C) and elective procedures only (D, E, F). These include Kaplan-Meier survival curves after cardiac surgery (A, D), mortality calculated for each month following surgery (B, E) and mortality six months after surgery (C, F). Expected mortality was calculated for age and gender-matched UK population. Mortality within the first six months after surgery is 33–42% higher than the mortality at 30 days from surgery or within the same hospital stay (i.e. the follow-up period that STS or EuroScore account for). The absolute excess mortality, above the one predicted by scores, is highest in patients in whom the risk predicted by mortality scores is high.

During a median follow-up of 6.2 years [2.8–11.3] and a cumulative follow-up period of 415,322.6 years, 5,926 patients died. There were 1,499 deaths within the first month after cardiac surgery, corresponding to a mortality of 2.61% (95%CI 2.49–2.75%). The 6-month mortality after cardiac surgery was 4.23% (95%CI 4.06–4.39%), while over the next five months (6^th^ to 11^th^ months after surgery) the mortality was only 0.57% (0.50–0.63%), suggesting increased mortality between the first and sixth month following cardiac surgery. Out of the 922 patients who died after the first, and before the sixth month after surgery, only 301 died without being discharged after surgery, while over two thirds (621) of deaths occurred after hospital discharge, and did not account for post-operative mortality, according to many surgical mortality risk scores, such as the EuroScore and the STS score (Fig 3).

Seven months after surgery, mortality appears to fall to a low and, thereafter, stable rate; when comparing the ratio of patients dying within each month to the number of patients at risk each month, the sixth month after surgery is the first month with mortality not differing significantly compared to the seventh month after surgery.

**Fig 4:**
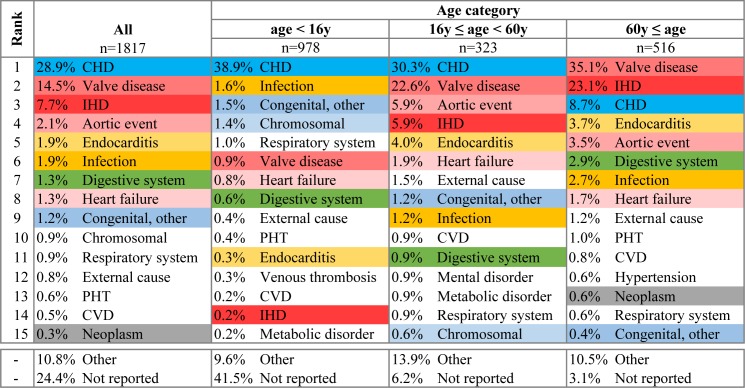
Leading cause of death in patients dying within 30 days from surgery or within the same hospital stay. Causes of death for patients who died within 30-days from surgery or within the same hospital stay. CHD, congenital heart disease; Congenital other, non-cardiac congenital malformation; IHD, ischemic heart disease; Aortic event, aortic aneurysm or dissection; PHT, pulmonary hypertension; CVD, cerebrovascular disease; HTN, systemic hypertension. Infection, not including endocarditis. The most common 10 causes of death are colour coded.

**Fig 5:**
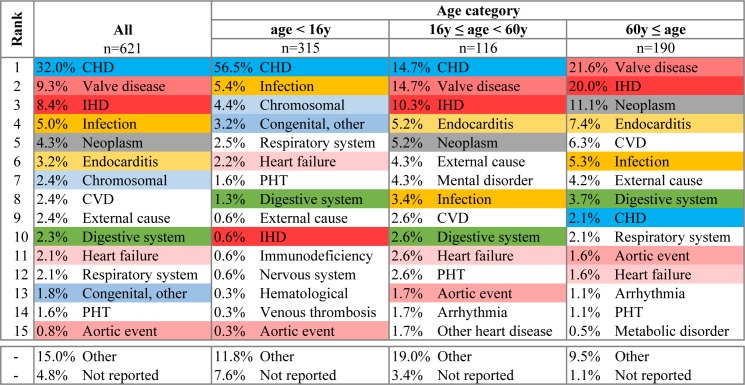
Leading cause of death in patients who died beyond 30 days from surgery and after discharge from hospital. CHD, congenital heart disease; Congenital other, non-cardiac congenital malformation; IHD, ischemic heart disease; Aortic event, aortic aneurysm or dissection; PHT, pulmonary hypertension; CVD, cerebrovascular disease; HTN, systemic hypertension. Infection, not including endocarditis. The most common 10 causes of death are colour coded.

Figs 4 and 5 show the leading causes of death after cardiac surgery. While in younger patients (<60y) the death was commonly attributed to CHD, in older patients (≥60y) the most common causes of death was valvular or ischemic heart disease. Endocarditis and other infections were infrequent causes of death in patients dying within 30 days after surgery or within the same hospital stay (1.9% for both), while contributed to death in a higher proportion of patients who died after discharge but within the first 6 months from surgery (5.0% and 3.2%, respectively, P<0.001).

### Predictors of mortality

**Fig 6:**
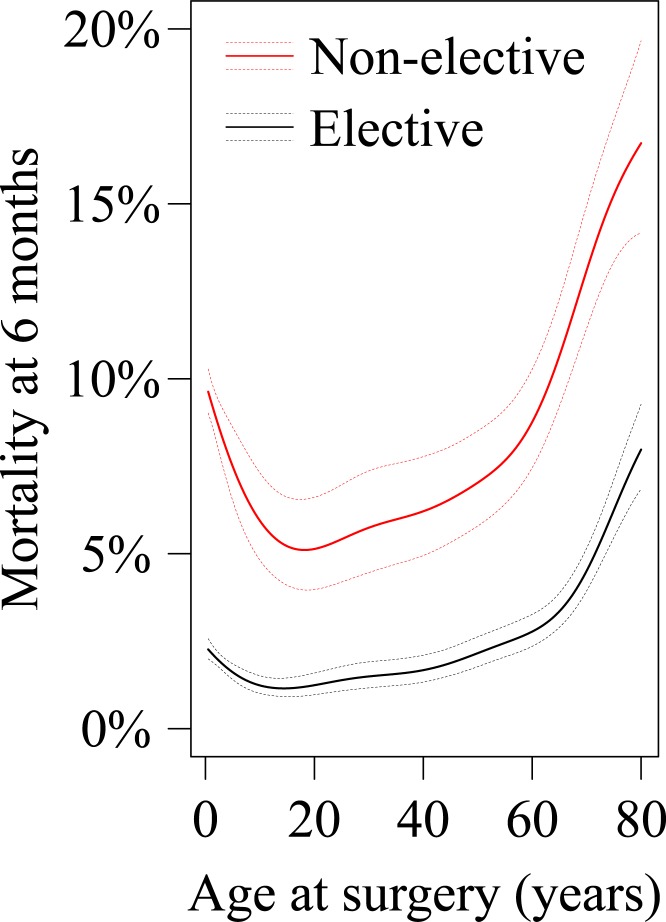
Mortality at six months after cardiac surgery in CHD patients. Mortality risk at 6 months after elective and non-elective cardiac surgery in CHD patients. There is a non-linear relation between age at operation and mortality, with a relatively sharp decline from birth to teenage years, with nadir at 15–18 years, slow increase in mortality until the age of 55–65 years, when mortality starts to rise sharply.

Several parameters were significantly associated with mortality at 6 months after cardiac surgery (Table 3, S4 Fig). The risk of death decreased rapidly with age in children (OR 0.18 per 10years, 95%CI 0.14–0.23, P<0.001, Fig 6), increased with a low slope in patients aged 15–60 years (OR 1.20 per 10 years, 95%CI 1.12–1.29, P<0.001), with further, significant increase in mortality risk with age in seniors (OR 1.65 per 10 years in patients above 60 years, 95%CI 1.48–1.85, P<0.001). On sensitivity analysis, after exclusion of operations including CABG or mitral valve surgery, there was similar excess of mortality at 6-months compared to 1-month as on the analysis of the entire dataset. Moreover, there was similar, J-shaped relationship of mortality at 1-month and at 6-months after surgery with highest mortality in young children and in seniors. Gender did not appear to influence perioperative mortality, while social deprivation was associated with increased mortality risk. The risk was also higher in patients with diabetes mellitus, systemic hypertension and in patients undergoing complex surgery (OR 1.32, 95%CI 1.21–1.44, P<0.001) or non-elective surgery (OR 4.15, 95%CI 3.82–4.50, P<0.001). On multivariable analysis, stratified for age, non-elective surgery (OR 4.16, 95%CI 3.81–4.54, P<0.001), diabetes mellitus (OR 1.34, 95%CI 1.12–1.61, P = 0.001), systemic hypertension (OR 1.14, 95%CI 1.01–1.30, P = 0.049) and white ethnicity (OR 0.77, 95%CI 0.70–0.85, P<0.001) were significantly associated with mortality.

**Table 3:** Results of univariable logistic regression analysis for mortality at 6 months from surgery.

Parameter	Odds ratio (95% CI)	P-value	
Age, per 10 years			
*Children*, *age ≤15y*	0.18 (0.14–0.23)	<0.001	*
*Adults*, *age 15-60y*	1.20 (1.12–1.29)	<0.001	*
*Adults*, *age 60y<*	1.65 (1.48–1.85)	<0.001	*
Female gender	1.07 (0.99–1.16)	0.10	
White ethnicity	0.80 (0.73–0.87)	<0.001	*
Social affluence‡	0.94 (0.90–0.98)	0.006	*
Diabetes mellitus	2.02 (1.72–2.37)	<0.001	*
Hypertension	1.47 (1.33–1.61)	<0.001	*
Obesity	1.09 (0.87–1.37)	0.45	
Renal failure	1.16 (0.99–1.35)	0.06	
Non-elective surgery	4.15 (3.82–4.50)	<0.001	*
Complex surgery	1.32 (1.21–1.44)	<0.001	*

The relation of age to six-month mortality was calculated separately for children (≤15y), adults (15-60y] and seniors (60y<).

(‡) For social affluence, the odds ratio was calculated for the z-score of Index of Multiple Deprivation Rank; a lower rank indicates increased deprivation. Complex surgery was defined as, more than one major procedure performed during the surgery.

(*) indicates statistical significance (P<0.05).

## Discussion

We report herewith outcomes for all cardiac surgery performed in patients with CHD within the NHS England, over the past 18 years. Our data show increasing surgical volume during the study period across age groups, particularly in adults. We demonstrate a U-shaped relationship between surgical mortality and age. Post-operative mortality remains elevated for up to 6 months after cardiac surgery.

### Increasing volume of cardiac surgery

Despite a relatively modest increase in England’s population between 1997 and 2014 (from 48.7 to 54.3 million, +11.5%) and relatively stable numbers of live births (from 609 to 661 thousand, +8.5%), there was a threefold increase in the number of cardiac procedures performed in patients with CHD (Fig 1, S1 Fig) [14]. The increase was evident in all age groups, with a twofold increase in patients under 10 years old and a six-fold increase in patients above 60 years old. The reason for this appears multifactorial. The prevalence of CHD diagnosed at birth appears to have increased over the study period only by approximately 30% [15–18]. The major factor contributing to the increasing surgical volume appears to be the substantial increase in lifespan for patients with CHD and, thus, a gradual increase in the number of CHD patients in the population [19,20]. Moreover, many patients require either percutaneous or surgical interventions later in life, which explains the significant increase of surgery performed from adolescence onwards. In fact, the increase in surgical volume continues, despite the introduction and increasing use of percutaneous interventions for ASD or VSD closure, stenting for aortic coarctation and percutaneous pulmonary or aortic valve implantation [21–24]. We submit that the increase in surgical volume is likely to continue, mostly in adult patients. The fiscal and infrastructure implications are obvious; there will be need for forward planning to increase the number of appropriately trained cardiac surgeons and cardiologists.

### Accounting for post-operative mortality

Current mortality risk stratification scores account for mortality related to cardiac surgery only if death occurs within 30 days from surgery or if the patient dies within the same hospital stay [2–7]. Similarly, the British National Institute for Cardiovascular Outcomes Research accounts for post-surgical death only if it occurs within 30 days of surgery [25]. The information derived from these scores is important for several reasons. It is used both for clinical decision-making and for consenting patients. Most of all, it is one of the most important measures of hospital quality care. This information is publicly available both in the UK and in the US and impacts directly on patients’ choice, both whether to undergo surgery and which surgical centre to choose. We demonstrate in our study that post-surgical mortality is 30 to 40% higher than estimated by the current mortality risk stratification scores which accounts for 30 days or index hospital stay mortality only; this is 40 to 50% higher compared to the 30-day mortality. This is particularly important in patients who have a relatively high absolute risk as estimated by current risk stratification methods, e.g. elderly patients. In such cases, the potential survival and clinical benefits need to be weighed carefully against the 6-month, and not the 30-day perioperative mortality risk. Percutaneous interventions, which are typically associated with a lower immediate post-operative risk, may thus need to be considered in a higher proportion of patients. For example, current guidelines suggest performing a transcatheter aortic valve replacement (TAVR) instead of surgical aortic valve replacement in patients with a logistic EuroSCORE >20% or STS score >10% [26,27]. If these scores “underestimate” procedure-related mortality by 35%, the cut-off for considering TAVR should be 15% for the EuroSCORE and 7% for the STS score. As the distribution of mortality risk in patients requiring aortic valve replacement is positively skewed towards a lower mortality risk [28], the above would result in a substantial increase in the number of patients eligible for TAVR.

The need for extended follow-up after general cardiac surgery, in general, has previously been pointed out by Siregar at al [29]. They analyzed outcomes after cardiac surgery in adults from 10 out of 16 cardiothoracic centres in Netherlands between 2007 and 2010. The majority of interventions were CABG, followed by valvular procedures. In their analysis, the post-surgical mortality was increased up to the 120^th^ day after surgery and they suggested accounting for mortality within this extended follow-up period after surgery. In contrast, in our study the mortality was increased up to the 180^th^ day after surgery, which may be associated with higher complexity of interventions in the setting of CHD.

### Predictors of post-operative mortality and cause specific mortality

Several parameters emerged as significant predictors of post-surgical mortality; particularly the relationship of age and type of surgery to mortality is interesting from a clinical perspective. Mortality risk declined with age in children, with a nadir at 15-18years for both elective and non-elective surgery; it increased at a slow rate with age in adults, until the 60^th^ year of life and then increased at a fast rate in seniors. Moreover, the risk of non-elective surgery increased in adults significantly with age. Thus, a proactive approach towards elective surgery may be appropriate in selected patients, particularly in patients approaching 60 years of age or older. Complex surgery was associated with a 30% higher risk of mortality. This association appears at least partially causative, as complex surgery may be associated with longer cardiopulmonary bypass time and higher complication rates. A staged, hybrid approach, addressing all amenable lesions percutaneously and focusing during cardiac surgery only on lesions that cannot be addressed percutaneously, may reduce mortality, although this is speculative.

The causes of death within the early post-operative period and the extended period, up to six months after surgery, are different for children and adults. Valvular causes, not specified as infection, are the leading cause of post-operative mortality both in the early and extended period in adults, but not in children. This reinforces the need for appropriate, post-operative follow-up for early detection and treatment of valvular dysfunction in adults. Interestingly, the contribution of infections to mortality, including endocarditis, does not decline after surgery. In children, infections, in general, were the cause of death in only 1.6% of cases in the early post-operative period but for 5.4% in the extended period. Moreover, the contribution of endocarditis to mortality increases with age, both in the early and extended post-operative period. This highlights the need for endocarditis prophylaxis in the broad sense. It should include encouraging meticulous dental hygiene, smoking cessation, appropriate skin hygiene avoiding unnecessary injuries, like tattooing or piercing, but also antibiotic prophylaxis in high-risk patients [30].

### Limitations

The vast majority of cardiac surgery performed in England is performed within the NHS; however, some surgery is privately funded and, thus, these data are not available in the NHS database. Patients with private insurance usually do no change to NHS and, conversely, most patients undergoing procedures under NHS usually continue with the national system. As a small proportion of patients may, however, have undergone private cardiac surgery within the study period, we decided not to analyze the crude number of previous cardiac surgery as a variable in the logistic regression model for post-operative mortality. Data are collected for the NHS database by trained and accredited coders. Although subject to error, this database was demonstrated to be more reliable than, for example, a dedicated national vascular surgery database [31]. Moreover, as the data were not specifically collected for this study, there was no selection bias.

## Conclusions

We demonstrate a significant increase in volume of cardiac surgery performed in CHD patients in England between 1997 and 2014 across all age groups, albeit this increase was greater in adults. Post-surgical mortality remained elevated for up to 6 months after cardiac surgery and surgical mortality risk stratification scores should account for this. The relationship of mortality and age was U-shaped, with mortality being highest in young children and in adults above 60 years.

## Supporting information

S1 Fig Number of cardiac operations relative to 1997. There was a significant increase in the number of annually performed cardiac operations between 1997 and 2014 for all patients and in all age categories. The increment of surgical volume was higher in older patients.
(EPS)

S2 Fig Percentage of complex surgery (more than one surgical procedure performed). The proportion on complex surgical procedures was lowest in patients aged 10–20 years (13%), with an increase in older patients. Moreover, there was an increase in the proportion of complex procedures over the years, from 14% in 1997 to 31% in 2015. This table presents data for the last cardiac surgery performed in each patient.
(EPS)

S3 Fig Percentage of non-elective surgery. The percentage of non-elective cardiac surgery was relatively stable over the years (22–30%). It was highest in children under the 10th year of live, was lowest in patients aged 10–20 years and gradually increased in adults. This table presents data for the last cardiac surgery performed in each patient.
(EPS)

S4 Fig Risk of death within 6-months after surgery by age category and main surgical procedure. This table presents data for the risk of death within 6 months from surgery analysed using logistic regression. Results are presented as odds ratio (OR) and 95% confidence intervals. Analysis for each category was performed only if there were at least 10 deaths in the model and at least 100 procedures in both comparison groups. ASD, atrial septal defect; VSD, ventricular septal defect; ToF, tetralogy of Fallot; AVSD, atrioventricular septal defect; CABG, coronary artery bypass graft; TGA, transposition of the great arteries; TAPVC, total anomalous pulmonary venous connection; TCPC, total cavopulmonary connection; AP Fontan circulation, atrio-pulmonary connection; DORV, double outlet right ventricle; TGA arterial switch—arterial switch for TGA or double switch for 'congenitally corrected' TGA.
(EPS)
